# Spatial Difference and Convergence of Ecological Common Prosperity: Evidence from the Yellow River Basin in China

**DOI:** 10.3390/ijerph20043370

**Published:** 2023-02-14

**Authors:** Pei Liu, Jiajun Xu, Xiaojun Yang

**Affiliations:** 1 School of Economics, Zhengzhou University of Aeronautics, Zhengzhou 450046, China; 2 Post-Doctoral Moving Station of Applied Economics, Henan University, Kaifeng 475004, China; 3 School of Economics and Management, Wuhan University, Wuhan 430072, China; 4 School of Economics, Zhongnan University of Economics and Law, Wuhan 430073, China

**Keywords:** ecological common prosperity, Yellow River Basin, spatial difference, convergence

## Abstract

Analyzing the spatial difference and convergence of ecological common prosperity (ECP) in the Yellow River Basin (YRB) will be beneficial for the environmental governance and multi-regional economic coordination. Based on the panel data of 97 cities in the YRB from 2003 to 2019, this paper measured and analyzed the index of ECP, the Gini coefficient of ECP, and the convergence of ECP. The results indicate that the ECP of YRB shows a steady growth trend (with an average growth rate of 4.71% yearly) and the overall differences are low (average Gini coefficient is 0.1509 from 2003 to 2019). In different areas, the Gini coefficient between the medium-stream and downstream of YRB is the largest (average value of Gini coefficient is 0.1561). From the decomposition of the overall differences of ECP, the contribution degree of the density of transvariation is the highest for annual average, with a contribution rate of 43.37%, the rate of intra-regional and the inter-regional differences are 31.86% and 24.77%, respectively. The results indicate that the overall differences of ECP in YRB are getting smaller because of cooperation and governance, but the differences between and within regions exist because of geographical feature. There is a significant spatial *β* convergence trend of ECP, the convergence rate in the upstream and downstream area is faster under the economic geographical matrix than others, and the rate in the medium-stream area is faster under the administrative adjacency matrix. Therefore, strengthening economic and environmental cooperation between and within regions is more beneficial to achieve a better quality of life, as well as the long-term goals of 2035.

## 1. Introduction

The Yellow River is an important and well-populated area of human habitation, and it is a rich water source region for north China, the second largest river in China, and the fifth largest river all over the world (Chen et al., 2020; Xu et al., 2009) [1,2]. Because of the process of social civilization, the human–land system, water resource, climate change, and ecological environment at the YRB have been affected by human activities (Jiang et al., 2021) [3]. Some problems of ecological destruction and environmental pollution are occurring and being experienced in many industrial regions of the Yellow River. In order to ensure a stable high-quality economy and sustainable development, *The Ecological Protection and High-quality Development of the YRB* (*EPHD of YRB*, *proposed in September, 2019*) in China as a national strategy was proposed (Jiang et al., 2021; Shi and Wang, 2021) [3,4]. Therefore, keeping coordinated and sustainable development of the human–land system in YRB is conducive and important for promoting ecological protection, ecological security, and high-quality development (Shi and Wang, 2021) [4]. It also has theoretical value and practical significance for the government to coordinate the relationship in policy and institution between sustainable economic development, resource consumption and protection, ecological security, and environmental protection, as well as to explore new ecological development models between different provinces and regions in the future (Jiang et al., 2021) [3].

At the same time, the Chinese government pointed out the long-term goals of socialist modernization in 2035, “The ecological environment will be fundamentally improved, and the goal of building a beautiful China will be basically realized” and “The people’s life will be better, the comprehensive development of human and the common prosperity of human will make more significant progress” (*Fifth Plenary Session of the 19th Central Committee of the Communist Party of China, 2020*). It shows that the ecological civilization goal for a better environment and the common prosperity goal for a better high-quality of life are the core of the new development stage in China, and they are also the key to reducing carbon emissions in achieving the goal of carbon peak by 2030, as well as the goal of carbon neutrality by 2060 under the threat and pressure of global climate change. The concept of ecological common prosperity in this paper just reflects the above goal contents, and the meaning of ECP also reflects the high coordination of economic value, ecological value, and human value, required by the development goal, which makes it valuable to analyze the index system of ECP in YRB of China.

The meaning of ECP is not only the common prosperity of private and family income in goal of economic development, but also the common prosperity of public goods and services, which include the ecological production, healthy environment, beautiful natural scenery, etc. It refers to the supply of natural and social ecological productions, required by people to maintain survival and sustainable development, which can not only be met in quantity, but also has a high level of quality. The contents of ECP involve the sustainability in economic development, the improvement in ecological environment quality, the greenization in production and lifestyle, the high efficiency in energy, and the security in ecology. The YRB is a major area in China shown above, so it is necessary to measure the index of ecological common prosperity and analyze the spatial difference and convergence of ECP in the YRB for the goal of economy, ecology, and society.

Therefore, the contribution of this paper is that it explains the meaning of ecological common prosperity (ECP) in detail based on the meaning of material common prosperity and spiritual common prosperity (*elaborated in the People’s Daily, 8 October 2021*), and then it establishes the index system of ECP and calculates it by the entropy method. Second, it chooses the area of YRB, where the national strategy was implemented (*EPHD of YRB, 2019*); YRB was also the place where the concept of ECP was practiced. Third, it analyzes the spatial difference of ECP by Gini coefficient and the convergence of ECP by spatial panel model, and more information about ECP is provided for high-quality and sustainable development.

## 2. Literature Review

The *EPHD of YRB* in China is a national and regional development strategy that started in 2019 (Yuan X et al., 2021) [5], so there are a large number of studies involving the YRB (Jiang et al., 2015; Liu et al., 2022; Shi and Wang, 2021; Wang et al., 2021; Wei et al., 2021; Xu and Song, 2022; Xu et al., 2009; Yuan et al., 2020; Yuan et al., 2022; Zhai et al., 2021; Zhang and Xu, 2022) [2,4,5,6,7,8,9,10,11,12,13]. Studies of the YRB mainly focused on the ecological environment, water resource, agriculture, climate change, carbon footprint, etc., such as the irrigation water rebound effect in YRB (Xu and Song, 2022) [10]: the irrigation water rebound effect included four parts, the effect in product income, product substitution, factor income, and the factor substitution. Liu et al. (2022) [7] analyzed the meteorological factors and water requirements of four crops in the YRB from 1971 to 2017, as well as the characteristics in spatio-temporal variation. Yuan et al. (2022) [5] analyzed the carbon footprint and estimated the amount of embodied carbon transfer between provincial regions and industrial sectors in YRB by the model of the multi-regional input–output, they found that the carbon footprint of the YRB from 2012 to 2017 declined by 23.4%.

However, the region of YRB is a complex eco-system; a coordinated development system is conducive to promoting ecological protection (*ecological civilization for a better environment of life*) and high-quality and sustainable development (*common prosperity in development for a better quality of life*) in YRB (Shi and Wang, 2021) [4]. The study of ecological civilization in YRB has mainly focused on water source and water management (Liu et al., 2022; Xu and Song, 2022) [7,10]; carbon footprints (Yuan et al., 2022) [5]; water footprints (Feng et al., 2012) [14]; efficiency in carbon emission (Zhang and Xu, 2022) [13]; response of streamflow to climate change (Xu et al., 2009) [2]; the dynamics of vegetation and the factors’ contribution under climate and non-climate (Yuan et al., 2020) [11]; spatio-temporal analysis of vegetation variation (Jiang et al., 2015) [6]; the coordinated development system between the human and land (Shi and Wang, 2021) [4]; and efficiency evaluation and efficiency influencing factors in agricultural water (Wei et al., 2021) [9]. The study of common prosperity in high quality before mainly focused on widening income disparity (Chang, 2002) [15]; economic inequality (Wu, S.X. et al., 2022; Zhang et al., 2020) [16,17]; income share and the Gini coefficient (Alvaredo, 2011) [18]; regional income and net wealth inequality (Song, 2013) [19]; measurement and analysis of equalization in public services (Liu and He, 2019) [20]; and the monitoring research on the index of common prosperity (Lyu and Chen, 2022) [21]; but there are few papers analyzing the coordination of ecological civilization and common prosperity in the YRB.

There are some scholars that have started to study the coordinated development level of YRB. Li et al. (2021) [22] found that the coupling coordination degree of production, life, and ecology in the YRB was relatively low, and a pyramid-shaped distribution was shown from 1995 to 2015. The YRB is an important area in water and energy, which is crucial for economic transformation (Zhao et al., 2021) [23] and the quality and quantity of the water resource (Chen et al., 2020) [1], but the risk in sustainability of the water resource and green development still exists, so it is very necessary to explore the coordinated development level and the sustainable development ability in YRB (Chen et al., 2020; Zhao et al., 2021; Li et al., 2021) [1,22,23]. Li et al. (2012) [24] found that a U-shaped curve was shown between urbanization and the environment by panel data and a coupling coordination model. Therefore, it is important in YRB to measure the coordinated development level, explore the spatial differentiation, analyze the influencing factors, and pursue a sustainable development pattern (Jiang et al., 2021) [3], which will be helpful for future planning of industrial layout, economic management and environmental governance, ecological restoration and protection, and high-quality living space (Li et al., 2021) [22].

In addition, the situation of industrial and energy structure, water resource, geographical slope, sunshine duration, regional temperature, the quantity of population, and the culture and lifestyle in different areas of the YRB is also different. Therefore, it is suitable and necessary to understand the dynamics and changes in YRB from a spatial view (Wang et al., 2010) [25], such as spatial spillover and spatial agglomeration. Gong et al. (2022) [26] found that spatial spillover and agglomeration effects in carbon emissions are obvious. Shi and Wang (2021) [4] found that the growth rate of residential land area in different reaches of the YRB kept a spatial heterogeneity: the growth rate in the upper reaches was higher, with a lower level in the medium and lower reaches, and the negative relationship between residential area and population was a commonly existed phenomenon. Moreover, efficiency of carbon emission was also significantly different between different provinces in YRB (Zhang and Xu, 2022) [13], a fluctuating upward trend was shown.

According to the above analysis, as the core contribution of this paper, it is necessary to build the index system and measure the index level of ECP (*ecological common prosperity includes the common prosperity in economic and ecological civilization in environment*), and then analyze the spatial difference and convergence of ECP in the different areas of YRB. It will be helpful to provide more facts to strengthen the coordinated development level of economy, ecology, and society between different regions of YRB according to the results for the final goal of *EPHD of YRB*.

## 3. Methodology and Data

### 3.1. Study Area

**Figure 1:**
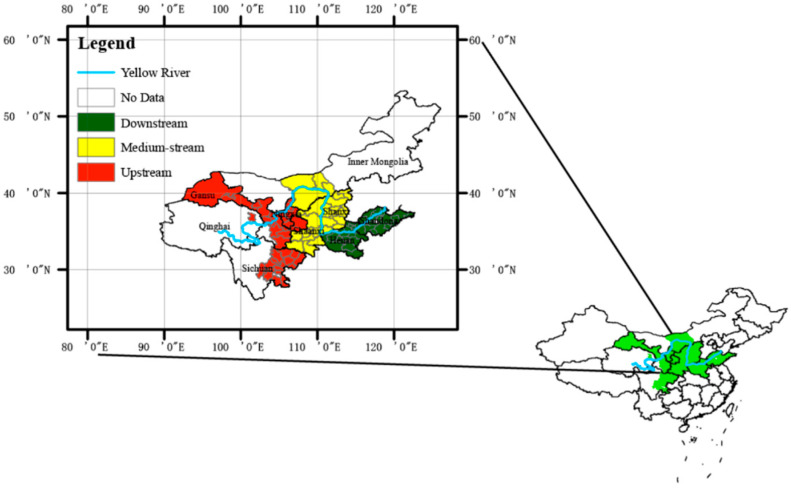
The location and the area (upstream, medium-stream, downstream) of Yellow River Basin.

The Yellow River Basin (shown in Figure 1) is located in 30–50° N and 96–119° E of the Chinese northern part (Li J, et al., 2021) [22], 1900 km from west to east and 1100 km from north to south, the area covers 1.3 million km^2^. The Yellow River Basin is the second largest river in China, at 5464 km in length, and is also the fifth largest river all over the world, the flow area contains the provinces of Inner Mongolia, Qinghai, Ningxia, Sichuan, Gansu, Shanxi, Henan, Shanxi, and Shandong. The total population of YRB was about 160 million at the end of 2019. Based on the file of *Outline of the Plan for EPHD of YRB*, the YRB was divided into three regions, including upstream, medium-stream, and downstream according to the natural environment and hydrological conditions of the area where the river flows, such as water, slope, temperature, human activities, etc. Moreover, some authors [2,5,9,10,22] who have studied the YRB also divided it into three regions. From the source of the river in Qinghai province to Hekou town in Inner Mongolia is the upstream of YRB. The middle-stream is from Hekou town to Taohuayu in Henan province. From Taohuayu to the estuary is the downstream of YRB.

### 3.2. The Measurement of ECP

#### 3.2.1. Standardize the Original Data

To measure the index of ECP and eliminate the heterogeneity of different data in magnitude and dimension, standardizing the original index is necessary (Chen et al., 2021) [27]. Based on the idea of confidence interval and quantile, the formula of data standardization is transformed as follows:

(1) $$y_{ij}=\frac{x_{ij}-x_{min}}{x_{1}-x_{min}}*0.1\;;\;x_{min}\leq x_{ij}<x_{1};\;0\leq y_{ij}<0.1$$

(2) $$y_{ij}=\frac{x_{ij}-x_{1}}{x_{9}-x_{1}}*0.8+0.1\;;\;x_{1}\leq x_{ij}\leq x_{9};\;0.1\leq y_{ij}\leq 0.9$$

(3) $$y_{ij}=\frac{x_{ij}-x_{9}}{x_{max}-x_{9}}*0.1+0.9;\;x_{9}<x_{ij}\leq x_{max};\;0.9<y_{ij}\leq 1$$

where $y_{ij}$ and $x_{ij}$ refer to the standardized data and the original data index; $x_{max}$ refers to the maximum value and $x_{min}$ refers to the minimum value of the original data; $x_{9}\;and\;x_{1}$ refer to the 10% quantile and 90% quantile.

#### 3.2.2. Calculate the Index

The index of ECP was calculated by the entropy method, shown as follows (Lyu and Chen, 2022; Tian and Wang, 2022; Wu, B. et al., 2022) [21,28,29]; where $e_{j}$ refers to the information utility value of the index $y_{ij}$; where $w_{j}$ refers to the weight of index $y_{ij}$; according to the index system of ECP, the entropy method in this paper is used to calculate the weight of variables, and evaluate the indicators under the comprehensive level. The index of D is the final composite index for multiple variables, and the $y_{ij}$ is the original data of variables.

(4) Pij=yij∑j=1j=nyij;   ej=−(1lnn)∑i=1i=npijln(pij);   wj=(1−ej)/∑j=1j=n(1−ej)

(5) $$0\leq w_{j}\leq 1;\;\sum_{j=1}^{j=n}w_{j}=1;\;\;\;\;D=\sum_{j=1}^{j=n}w_{j}x_{j}$$

### 3.3. Dagum Gini Coefficient and Its Decomposition Method

In this paper, the index system of ECP was shown in Table 1, the detailed decomposition method of Gini coefficient, proposed by Dagum (1998), is used to reveal the regional differences of ECP in the YRB and the root causes of differences. ® Dagum Gini coefficient formula for measuring the index of ECP is set as follows:

(6) $$G=\sum_{j=1}^{k}\sum_{h=1}^{k}\sum_{i=1}^{n_{j}}\sum_{r=1}^{n_{h}}\left|y_{ji}-y_{hr}\right|/2n^{2}\mu$$

Among them, the index of *G* is the Gini coefficient; the indicator of *k* is the total number of YRB regions (*k* = 3); the *n* is the total number of YRB cities (n = 97); *n_j_(n_h_*)is the number of cities in the *j(h*)region; *y_ji_(y_hr_*)is the ECP level of city *i(r*) in the *j(h*) region, and *μ* is the average level of ECP.

According to the average development level of ECP in the region, the three regions are sorted, and the Gini coefficient *G_jj_* of region *j*, and the Gini coefficient *G_jh_* of region *j* and region *h* are calculated. The specific formula is set as follows:

(7) $$G_{jj}=\sum_{i=1}^{n_{j}}\sum_{r=1}^{n_{j}}\left|y_{ji}-y_{jr}\right|/2n_{j}^{2}\mu_{j}$$

(8) $$G_{jh}=\sum_{i=1}^{n_{j}}\sum_{r=1}^{n_{h}}\frac{\left|y_{ji}-y_{hr}\right|}{n_{j}n_{h}\left(\mu_{j}+\mu_{h}\right)}$$

According to the way of the Dagum Gini coefficient, the whole value of Gini coefficient *G* in YRB can be divided into three parts, intra-regional difference contribution (*G_w_*) of the overall Gini coefficient *G*, inter-regional net difference contribution (*G_nb_*) of the whole Gini coefficient *G*, and density of transvariation contribution (*G_t_*) of the overall Gini coefficient *G*. *D_jh_* is the relative influence of index level of ECP between regional j and h. The specific formula is set as follows:

(9) $$G_{w}=\overset{k}{\underset{j=1}{\sum }}G_{jj}p_{j}s_{j}$$

(10) $$G_{nb}=\overset{k}{\underset{j=2}{\sum }}\overset{j-1}{\underset{h=1}{\sum }}G_{jh}(p_{j}s_{h}+p_{h}s_{j})D_{jh}$$

(11) $$G_{t}=\overset{k}{\underset{j=2}{\sum }}\overset{j-1}{\underset{h=1}{\sum }}G_{jh}(p_{j}s_{h}+p_{h}s_{j})\left(1-D_{jh}\right)$$

(12) $$p_{j}=\frac{n_{j}}{n};\;s_{j}=\frac{n_{j}\mu_{j}}{n\mu }$$

### 3.4. Spatial Convergence Model

β convergence is a commonly used convergence method, which can be divided into two research methods, the one is absolute β convergence and another one is conditional β convergence, in which the conditional β convergence refers to the state of convergence under the condition of social and economic characteristics of the controlled area, which is more in line with the reality of economic development than absolute β convergence.

Therefore, this paper focuses on conditional *β* convergence. As the mobility of factor resources and spatial interaction between cities continue to increase, the development level of ECP will have the spatial dependence, and then a spatial panel model is established to measure the spatial heterogeneity and convergence characteristics of ECP in the YRB. The specific model of ECP convergence is set as follows:

(13) $$\ln\left(\frac{{\mathrm{ECP}}_{i,t+1}}{{\mathrm{ECP}}_{i,t}}\right)=\beta \ln{\mathrm{ECP}}_{i,t}+\rho \overset{N}{\underset{j=1}{\sum }}w_{ij}\ln\left(\frac{{\mathrm{ECP}}_{i,t+1}}{{\mathrm{ECP}}_{i,t}}\right)+\theta \overset{N}{\underset{j=1}{\sum }}w_{ij}\ln{\mathrm{ECP}}_{i,t}+\delta X_{i,t}\;+\gamma \sum_{j=1}^{N}w_{ij}X_{i,t}+\mu_{i}+\eta_{j}+\varepsilon_{i,t}\;;\;\;\varepsilon_{i,t}=\lambda \sum_{j=1}^{N}w_{i,j}\varepsilon_{j,t}+\sigma_{i,t}$$

Among them, ECP_i,t+1_ and ECP_i,t_ are the index level of ECP of the city i in the t+1 and t year, respectively. *β* is the convergence coefficient of ECP. If *β* (*β < 0 or β* ≥ *0*) is significantly negative (that is *β < 0*), it means that there is conditional convergence in ECP, and its convergence speed and ability can be represented as *v*, and $v=-\frac{\ln\left(1+\beta \right)}{T}$; *ρ* is the spatial regression coefficient of ECP; *λ* is the spatial error coefficient of ECP; *θ* is the spatial spillover coefficient of ECP; *W* is the spatial weight matrix of ECP in YRB; *X* is the model control variables; *δ* is the estimation coefficient matrices of all control variables; *γ* is the spatial estimation coefficient matrices of *X*; *μ* and *η* are the spatial effect and time effect, respectively; *ε* is the independent and identically distributed interference term, and *ε* is idd.

According to the steps proposed by Elhorst (2014) [30], LM statistics (Lagrange Multiplier test), Wald statistics (Wald test), and LR statistics (Likelihood Ratio test) are used to select the corresponding model types when it will select the spatial regression models. The Likelihood Ratio test (LR), Wald test (Wald)m and Lagrange Multiplier test (LM) are the three major tests of econometrics, which can be used to check whether the constraints we set are valid. The three test methods are fairly equivalent, but the test methods are different. The Likelihood Ratio test (LR) is used to estimate the likelihood function value of the unconstrained model and the constrained model, and it uses the ratio of the two to construct statistics for hypothesis testing. Wald test (Wald) is used to estimate the unconstrained model, construct statistics according to the constraint conditions, and conduct a hypothesis test. The Lagrange Multiplier test (LM) is used to estimate the model with constraints, establish auxiliary regression according to constraints, and construct statistics according to the determinable coefficients of auxiliary regression for hypothesis test. At the same time, in order to overcome the endogenous problems existing in the econometric model, two methods are adopted. Firstly, the data of explanatory variables in the model are all delayed by one year, so as to overcome the bidirectional causal relationship between explanatory variables and the growth rate of ECP. Secondly, the method of quasi-maximum likelihood estimator of Lee and Yu (2010) [31] is used to estimate the model effectively.

### 3.5. Spatial Weight Matrix

For the choice of the spatial weight matrix, four spatial weight matrices are constructed to ensure the reliability of the results and reduce errors of regression (Xu et al., 2022) [32]. The first spatial weight matrix of ECP is *W_d_* (*the geographical distance weight matrix*), which means the reciprocal of spatial geographical distance between two cities of YRB. The next kind of spatial weight matrix is *W_e_* (*economic distance weight matrix*), which means the reciprocal of the average level of real GDP per capita between the two cities from 2003 to 2019 (based on the year of 2003). The third spatial weight matrix of ECP is *W_a_* (*adjacent administrative weight matrix*), which is a binary matrix (*W_a_ = 1 or 0*) that is set and divided by the provincial regions, if two cities belong to one province of YRB, the value of matrix is 1(*W_a_ = 1*); otherwise, the value of matrix is 0 (*not belong to one province, W_a_ = 0*). The fourth spatial weight matrix is *W_de_* (*economic geographical distance nested matrix*), which is to explain the spatial connection between cities that is influenced by both geographical distance *W_d_* and economic distance *W_e_*. Here, the method of Shao et al. (2016) [33] is adopted to set the weight; the weight of *W_d_* is 0.5 and the weight of the *W_e_* is 0.5. The four kinds of matrices are as follows.

(14) $$W_{d}=1/d_{ij}\;\left(i\neq j\right)$$

where $d_{ij}$ refers to Euclidean distance between two cities *i* and *j* that was measured by latitude and longitude.

(15) $$W_{e}=\frac{1}{pgdp_{ij}}\;,\;pgdp_{ij}=0.5*\;\left[\;pgdp_{i}+pgdp_{j}\right]\;\left(i\neq j\right)$$

where $pgdp_{i}$ refers to the real value of GDP per capita in city *i* from 2003 to 2019, $pgdp_{j}$ refers to the real value of GDP in city *j*, $pgdp_{ij}$ is the average value.

(16) $$W_{a}=\left\{\begin{array}{c} 1,\;\;if\;i\;and\;j\;\in p\;\\ 0,\;\;if\;i\;and\;j\;\;not\in p \end{array}\right\}$$

where, if city *i* and city *j* belong to one provincial region *p*, the value of matrix is 1, otherwise, the value is zero.

*W_de_* = 0.5 * *W_d_* + 0.5 * *W_e_* (17)

where *W_de_* is the nested matrix, the weight of *W_d_* is 0.5 and the weight of the *W_e_* is 0.5. * represents a multiplication relationship.

### 3.6. Control Variables

Referring to scholars’ research on the influencing factors of ECP in the existing literature (Li et al., 2021; Xu et al., 2022) [22,32], the control variables selected in this paper include population density (*POP*), economic development level (*PGDP*), industrial structure (*IS*), specialized agglomeration (*SA*), diversified agglomeration (*DA*), environmental regulation (*ER*), scientific and technological investment (*TI*), human capital (*HC*), etc.

Among them, the density of population (*POP*) is calculated by the natural logarithm of the whole city population, divided by administrative area; level of economic development (*PGDP*) is calculated by the natural logarithm of real GDP per capita (based on 2003); the industrial structure (*IS*) is calculated and measured by the proportion of the added value of secondary industry to GDP; the indicators of specialized agglomeration (*SA*) and diversified agglomeration (*DA*) refer to the treatment method of Pei et al. (2021) [34]. The former, *SA*, is measured by the relative specialization index, and the latter, *DA*, is measured by the relative diversification index. The specific model is as follows.

(18) $$SA_{it}=\max_{j}\left(\frac{S_{ijt}}{S_{jt}}\right)$$

(19) $$DA_{it}=1/\sum_{j}\left|S_{ijt}-S_{jt}\right|$$

where *S_ijt_* represents the ratio of employees in industry *j*, city *i*, year *t* to the whole number of employees in city *i*, *S_jt_* represents the proportion of employees in industry *j* and year *t* to the whole employees in the country. The intensity and strength of urban environmental regulation (*ER*) is set by measuring and calculating the comprehensive index of environmental regulation based on the entropy method (Li and Zou, 2018) [35]. The specific system of indicators contains three parts, they are the rate of utilization in industrial solid waste (%), the rate of treatment in wastewater (%), and the rate of harmless treatment in domestic garbage (%).The investment in science and technology (*TI*) is measured by the proportion of scientific research expenditure to the public budget fee of local governments. Human capital (*HC*) is obtained by the natural logarithm of the number of students that was in colleges (ten thousand people). Data descriptions of above variables are shown in Table 2.

## 4. Spatial Difference of ECP in the YRB

### 4.1. Spatial Characteristics of ECP

**Figure 2:**
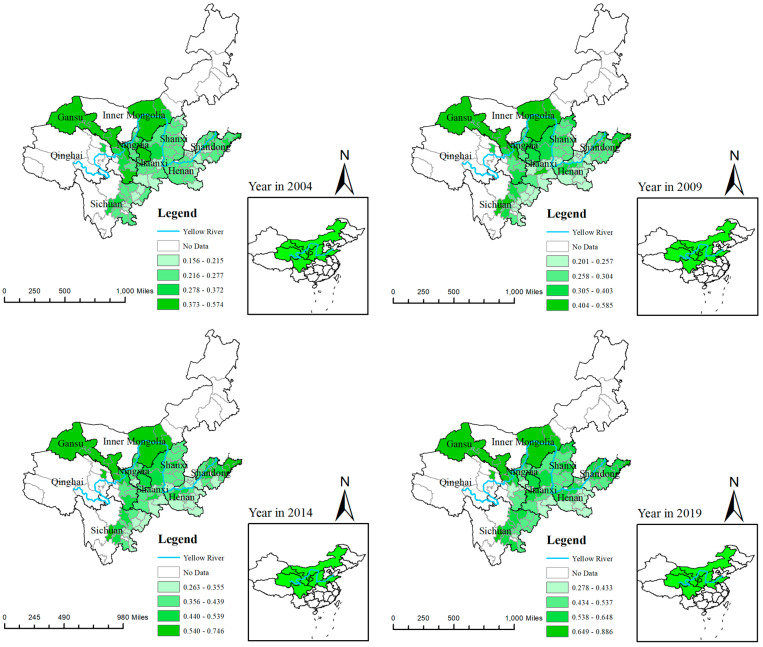
The spatial distribution map of ECP in YRB over the years in nine provinces.

Based on the index system of ECP in Table 1, using the panel data of 97 cities, the index level of ECP was calculated from the year of 2003 to 2019, and the spatial distribution characteristics of ECP in different cities and times is shown in Figure 2, which was drawn with ArcMap 10.2. We can know that high-value areas of the index of ECP were mainly distributed in the upstream area of YRB during different four years, such as Gansu, Inner Mongolia, and Ningxia. The main reason for this distribution is that the ecological environment and resources in upstream of YRB remain higher, and the industry resources are gathered in the downstream, which lead to waste discharge and environment pollution, as well as higher Engel’s coefficient in common prosperity, far from the concept of ecological common prosperity.

Comparing the spatial distribution in 2004 and 2019, the index of ECP in medium-stream and downstream of YRB in 2019 was higher than that in 2004, such as the cities in Shandong, Henan, Sanxi, Sichuan, and the minimum and maximum value in 2019 are higher than that in 2004, 2009, or 2014. This indicates that the whole level of ECP is constantly improving under the concept of ecological civilization in the road of common prosperity.

### 4.2. Evolution Trend of ECP in the YRB

**Figure 3:**
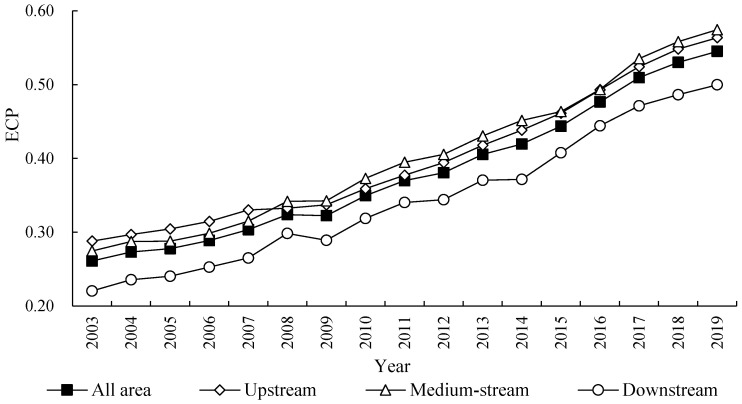
Evolution trend of ECP in the YRB from 2003 to 2019.

In order to reveal and analysis the evolution trend of ECP in the YRB during the inspection period, this paper calculates the index level of ECP in 97 prefecture-level cities of YRB from 2003 to 2019 based on the entropy method. The average level of ECP in the whole area of YRB, and average level in areas of upstream, medium-stream, and downstream are shown in Figure 3; it shows the evolution trend of ECP in the YRB from 2003 to 2019.

The results in Figure 3 show that the ECP in the YRB has some of the following kinds of characteristics. Firstly, the index level of ECP in the YRB shows a steady growth trend, with its index increasing from 0.2609 in 2003 to 0.5451 in 2019, with an increased ratio of 108.91% and an average growth ratio of 4.71% for every year, which expresses that the ECP has continuously improved. Secondly, the absolute difference in ECP between the areas of upstream, medium-stream, and downstream of YRB is small, but the range generally slowly expanded from 0.0676 in 2003 to 0.0747 in 2019. Specifically, the ECP in the medium-stream area of YRB is the highest, slightly higher than that of the upstream, but obviously higher than that of the downstream, which indicates that the ECP of the YRB is characterized by regional imbalance, and the ECP value of the medium-stream and upstream areas is obviously higher than that of the downstream. Third, there are some diversities in the growth ratio of ECP between the upstream, medium-stream, and downstream areas, among which the growth rate of downstream was fastest, with an increased ratio of 126.82%, and an average growth ratio of 5.25% for every year. Followed by the medium-stream, with an increased ratio of 109.21%, and an average increase ratio of 4.72% for every year. The growth rate in the upstream is the slowest, with a growth rate of 95.75%, and an average growth rate of 4.29% for every year, which shows that the ECP of all regions in the YRB has continuously improved.

### 4.3. The Overall Differences and Evolution Trend of ECP in the YRB

In order to further analyze the relative difference of ECP in YRB, according to the model of Dagum Gini coefficient and the method of decomposition, the regional difference, and their sources of ECP from year of 2003 to 2019 were calculated and further decomposed.

**Figure 4:**
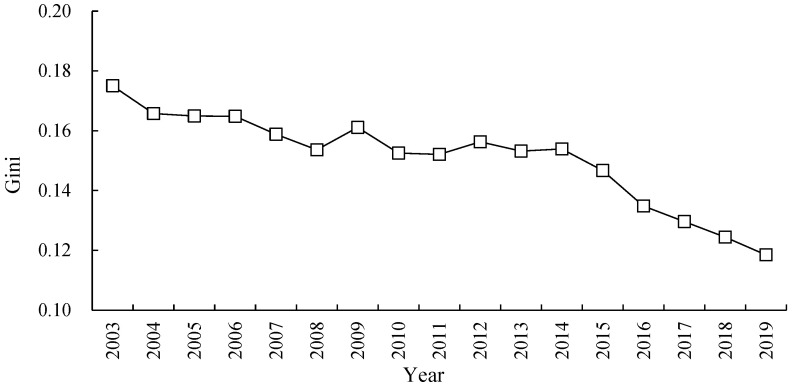
Overall difference and evolution trend of ECP in the YRB.

The result in Figure 4 depicts the overall difference and evolution trend of ECP in the YRB. It shows that the average Gini coefficient of ECP in the YRB is 0.1509 from year of 2003 to 2019, which reveals that the overall difference of ECP is low. It also shows an obvious downward trend in observation period, with an annual average decline rate of 2.41%. Specifically, the downward trend of the overall difference was relatively gentle from 2003 to 2014, with the Gini coefficient decreasing from 0.1750 to 0.1539 in 2003–2014, the average decrease is 1.16% for every year. The subsequent downward trend became very fast in 2014–2019, with an average decline rate of 5.09% from 0.1539 to 0.1185 in 2014–2019. Therefore, there are some differences for the ECP in YRB, and the degree of differences continues to weaken over time.

### 4.4. Intra-Regional Differences and Evolution Trend of ECP in the YRB

**Figure 5:**
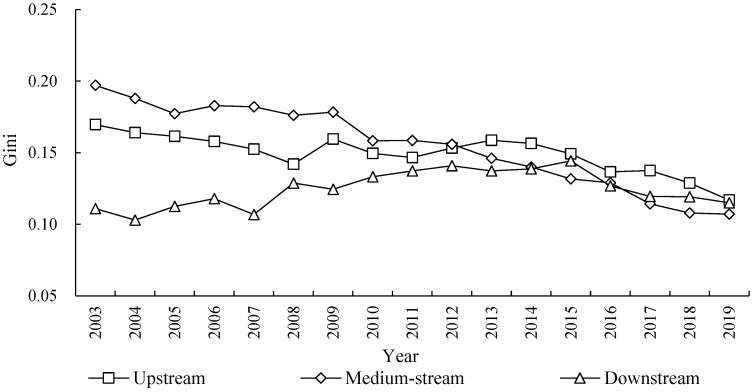
Intra-regional differences and evolution trend of ECP in the YRB.

The result in Figure 5 describes the intra-regional differences of ECP and shows its evolution trend in the YRB. From the value level of the Gini coefficient, the regional differences of ECP in the YRB from high to low are the areas of medium-stream, the upstream, and the downstream in turn. Specifically, the Gini coefficient of the ECP in the medium-stream is the largest from 2003 to 2019, the average value is 0.1548, that is higher than the overall value of the YRB. The Gini coefficient of the ECP in the upstream is large, the average value is 0.1495, that is lower than the overall value level of the river basin. The Gini coefficient of ECP in the downstream is small; the average value is 0.1245, far lower than the overall level of the YRB. This shows that the ECP level in the medium-stream has obvious spatial imbalance characteristics, while the spatial imbalance characteristics of the upstream and downstream cities are relatively small.

From the evolution trend, during the sample observation period of 2003 to 2019, the level of ECP in the medium-stream cities shows a continuous downward trend, and the Gini coefficient decreased by 3.74% annually, which indicates that the spatial imbalance of ECP in the medium-stream cities continues to improve. The changing trend of upstream cities is basically consistent with the overall difference of YRB, showing a downward trend, which indicates that the unbalanced phenomenon of ECP level in the upstream city has also been improved, but the improved speed is relatively slow, which is closely related to the small gap between the level of ECP in the upstream cities. The level of ECP in the cities of the downstream area shows an upward trend at first and then decreases, but it shows an upward trend on the whole. Its Gini coefficient fluctuates from 0.1109 to 0.1443 in 2003–2015, and it gradually decreases to 0.1150 in 2019, which indicates that the unbalanced phenomenon of ECP in the cities of downstream area continues to increase weakly, which is not conducive to the realization of the goal in ECP.

### 4.5. Inter-Regional Differences and the Evolution Trend of ECP

**Figure 6:**
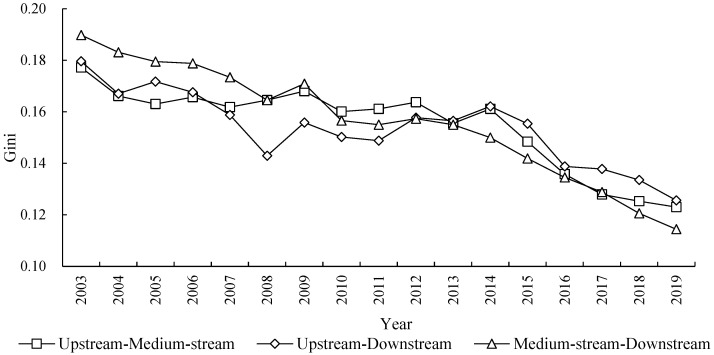
Inter-regional differences and evolution trend of ECP in the YRB.

The result in Figure 6 describes the regional differences and shows its evolution trend of ECP in the different areas of YRB. From the Gini coefficient value, the Gini coefficient values between the medium-stream and downstream area in the observation period are the largest, with an average value of 0.1561; and the average Gini coefficient is 0.1546 between upstream and medium-stream, and it is in a medium level. Finally, the average value of Gini coefficient between the upstream and downstream is lowest, and its value is 0.1535. This shows that there is a big regional gap between the medium-stream and downstream areas of the city, while the difference between the upstream area and downstream areas of the city is small.

From the Gini evolution trend, we can know that the Gini coefficient of ECP between different regions showed a continuous downward trend in the observation year, among which the Gini coefficient between medium-stream and downstream areas of the city showed the fastest downward trend, with the Gini coefficient dropping from 0.1898 to 0.1144 in 2003–2019, with a drop ratio of 39.73% and an annual average drop ratio of 3.11%. The Gini coefficient between upstream and medium-stream cities decreased from 0.1796 to 0.1230 in 2003–2019, the decrease rate is 30.59% and the average decrease rate is 2.26% for ever year.

Finally, the Gini coefficient between upstream and downstream cities dropped the lowest from 0.1772 to 0.1256 in 2003–2019, with a drop rate of 30.07% and an annual average drop rate of 2.21%. On the whole, the regional differences of ECP between different areas (among upstream, medium-stream, and downstream areas) in the YRB are weakening, and the unbalanced phenomenon of ECP is being improved, showing a convergence characteristic in ECP.

### 4.6. The Contribution Sources of Regional Differences of ECP

**Figure 7:**
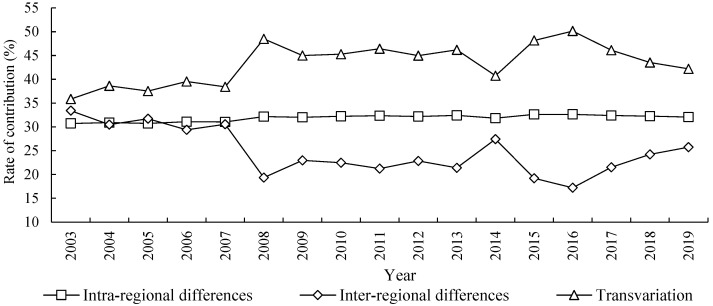
The contribution sources of regional differences and evolution trends of ECP in the YRB.

The result in Figure 7 depicts the regional difference sources and shows the evolution trend of ECP in the YRB. From the decomposition of the overall difference source, the contribution degree of density of transvariation is the highest in the observation period, and its contribution rate is between 35% and 51%, with an average ratio of 43.37% yearly. Secondly, the intra-regional difference of ECP is at a medium level, whose contribution rate is between 30% and 33%, with an average contribution rate of 31.86% yearly. Finally, the inter-regional difference is lowest, its contribution rate is between 17% and 34%, with an average rate of 24.77% yearly. This shows that the rank of whole differences of ECP in the YRB in order are from the density of transvariation, inter-regional differences, and intra-regional differences, among which density of transvariation is the main source of ECP differences.

From the time evolution trend, the contribution rate of intra-regional differences is relatively stable, showing a trend of “slightly rising-keeping stable”. At first, it slowly increased from 30.73% in 2003 to 32.16% in 2008, and then basically remained between 32% and 33%. The contribution rate showed a slight upward trend on the whole, the average increase rate is only 0.27% every year. Secondly, the contribution ratio in term of inter-regional differences showed a trend of continuous fluctuation, it dropped slightly from 33.41% in 2003 to 19.34% in 2008, then fluctuated to 27.42% in 2014, and dropped slightly to 17.21% in 2016. Finally, it rose slightly to 25.74% in 2019. The overall contribution ratio of inter-regional differences in 2003–2019 showed a slowly downward trend, with an average decline rate of 1.1% every year. Thirdly, the contribution rate of the density of transvariation also showed a trend of continuous fluctuation in Figure 7. At first, it rose slightly from 35.87% in 2003 to 48.50% in 2008, then the fluctuation dropped to 40.74% in 2014, and it rose to 50.15% in 2016. Finally, it remained at 42.19% in 2019. The overall contribution ratio of the density of transvariation indicated an upward trend, the average increase ratio was 1.02% every year.

On the whole, the fluctuation range of the contribution rate of intra-regional differences is very small, which indicates that the ratio of intra-regional differences on the whole remains relatively stable. The ratio of inter-regional differences in all differences is low, and the contribution ratio displays a downward trend, which shows that the ECP among different regions shows convergence characteristics over time, and the narrowness of the inter-regional differences is also a good and important phenomenon to promote the coordination of ecological civilization and common prosperity. The contribution ratio in the density of transvariation is the chief part of the overall differences of ECP in the YRB, and the contribution degree is always increasing, which indicates that the interaction effect between intra-regional and inter-regional differences is becoming the core element to influence the differences, and the coordination in intra-regions and inter regions has continuously increased, so we need to pay more attention on the coordination to overcome the differences of ECP.

## 5. The Spatial Convergence Analysis of ECP in the YRB

### 5.1. Spatial Correlation of ECP

To ensure the effectiveness of results in the empirical analysis by the spatial econometric method, firstly, Moran’s I index was used to assess whether the ECP has a spatial effect in the different regions of the YRB. Based on four kinds of spatial weight matrices, namely geographical distance weight matrix (*W_d_*), economic distance weight matrix (*W_e_*), administrative adjacent weight matrix (*W_a_*), and economic and geographical distance nested matrix (*W_de_*), the Moran’s I index of the ECP was calculated in the YRB from 2003 to 2019, and the results are displayed in Table 3.

The results showed that, except for the weight matrix of economic distance (*W_e_*) in 2003, the Moran’s I index of ECP in other spatial weight matrices was significantly positive each year, which revealed that there was a positive effect significantly and a spatial agglomeration effect in terms of correlation of ECP in the observation period. In addition, the spatial correlation under the administrative adjacent weight matrix (*W_a_*) was the strongest, while the spatial correlation under the weight matrix of geographical and economic distance (the matrix of *W_d_* and *W_e_*) was weaker.

From the evolution trend in time, under the matrix of geographical distance (*W_d_*), the Moran’s I index of ECP generally maintains a slow decline. From the perspective of the weight matrix (*W_e_*), the Moran’s I index of ECP generally maintains an upward trend. In the nested matrix of economic and geographical distance (*W_d_*_e_), the Moran’s I index of ECP generally keeps slowly rising. Under the matrix of administration adjacent weight matrix (*W_a_*), the Moran’s I index of ECP generally maintains a downward trend. This shows that the spatial dependence of ECP between economically adjacent cities will gradually increase with time, while the spatial dependence of ECP between geographically or administratively adjacent cities will gradually decrease with time.

### 5.2. Spatial Agglomeration Characteristics of ECP

**Figure 8:**
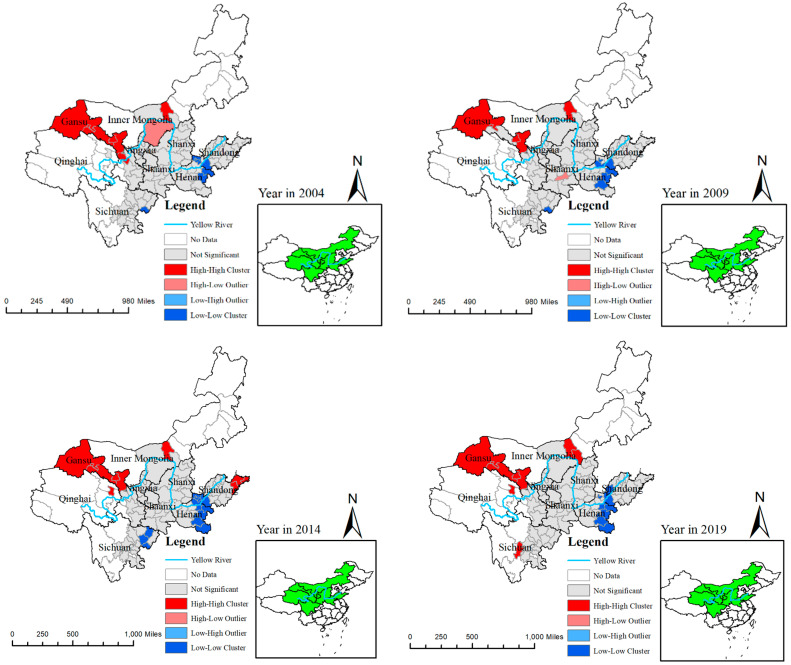
Spatial agglomeration characteristics of ECP in the YRB in different year.

For the analysis of spatial agglomeration characteristics of ECP, it uses local Moran’s I index of ECP to measure and analysis its spatial agglomeration; the distribution maps of the four years in 2004, 2009, 2014, and 2019 were drawn with ArcMap in Figure 8. The H-H cluster areas in the different years were mainly distributed in Gansu province and some area of Inner Mongolia, the L-L cluster areas were mainly distributed in some areas of Henan and Shandong Province, the areas of H-L and L-H clusters retained a low level, and the not-significant area was widely distributed in medium-stream and downstream areas of YRB. This indicates that the agglomeration level of ECP remained high in upstream, the medium-stream with a medium level, and downstream with a low level. This shows that the agglomeration level was related to the regional geographical ecological environment and development level of economic in YRB, and there was a significant spatial relationship in spatial regional distribution.

### 5.3. The Global Spatial Convergence of ECP

In this paper, the LM test, Wald test, and the LR test were used to choose the proper model, such as spatial lag model, spatial error model, or spatial Durbin model. All results in the test of significance passed at 1%, so the method of spatial Durbin model was used as the major model in the analysis of global spatial convergence, and the quasi-maximum likelihood estimation method was used to estimate the results of model.

Table 4 shows the results of analysis, which reported the estimation results of four different spatial matrices (*W_d_; W_e_; W_de_; W_a_*) by the model of spatial Durbin. The results show that the regression coefficients *β* of ECP in different spatial weight matrices are all negative significantly, and its significance was at the 1% level, which shows that the growth ratio of ECP in the YRB is negatively correlated with the initial development level; that is, there is a significant convergence trend of spatial conditions *β* in ECP. This means that, when considering the influencing factors of population density, economic development, industrial structure, agglomeration, environmental regulation, investment in science and technology, and human capital, the level of ECP will eventually converge to the same level, and it keeps a steady state over time.

In addition, the coefficient *ρ* from spatial regression and the coefficient *θ* from spatial spillover (except the coefficient *θ* in *W_e_* weight matrix) in all models are also significantly positive, which shows that there is a positive spatial relationship and a significant spatial spillover effect on the value of ECP in the YRB, that is, the ECP level of geographically or administratively adjacent cities has a remarkable promoting effect on local cities, which makes the gap between adjacent cities in ECP gradually narrow, which also confirms the reality that the development difference of ECP is gradually declining.

From the comparison of the different models, the convergence coefficient *β* of the ECP under the four spatial weight matrices has little difference, which makes the convergence speed difference smaller, being 2.32%, 2.46%, 2.61%, and 2.33% respectively, but the convergence speed under *W_a_* matrix is relatively fast. The above phenomenon shows that it is easy for administrative and economic neighboring cities to realize coordination development of ecological civilization and common prosperity with neighbors as partners. The reason may be that ecological civilization, common prosperity, and other factors have obvious spatial spillover characteristics, and the cities with similar provinces and economies usually have similar geographical advantages, which makes the level of ecological civilization and common prosperity spill over first in administrative and economic neighboring cities, thus making the administrative and economic neighboring cities realize coordinated development in ecological civilization and common prosperity.

### 5.4. The Regional Spatial Convergence of ECP

In addition to the above studies, this paper also discusses whether there is a convergence characteristic on spatial conditional *β* for the value of ECP in upstream, medium-stream, and downstream areas of the YRB. According to the model selection steps, all the test values passed through the test in significance by 5% at least. Finally, the spatial Durbin model was adopted as the major model in analysis of regional spatial convergence, and the method of quasi-maximum likelihood estimation was also used to assess the models under four different spatial weight matrices. The results are displayed in Table 5.

The results in Table 5 show that the coefficients *β* of ECP under four different spatial matrices (*W_d_; W_e_; W_de_; W_a_*) are all negative, and pass through the test of significance at 1%, which suggests that ECP has a significant convergence trend of spatial conditions *β* in upstream, medium-stream, and downstream areas of the YRB. The spatial regression coefficients *ρ* in all models are also positive, and they passed though the test of significance at 10% at least, which suggests that there is a positive spatial correlation significantly in ECP between upstream, medium-stream, and downstream areas of the YRB.

From the view of the spatial spillover effect, the spatial spillover coefficient *θ* of ECP in the upstream area of YRB is only significantly positive in the administration adjacent weight matrix *W_a_*, which shows that there is a remarkable positive effect on spatial spillover at the value of ECP among administratively adjacent cities in the upper reaches of YRB. The coefficient *θ* of spatial spillover effect on ECP in the medium-stream area of the YRB is all significantly positive, which shows that there is a positive remarkable spatial spillover effect on the value of ECP among adjacent cities in economic, geography, or administration from the medium-stream area of YRB. In the downstream area of YRB, except for the spatial spillover coefficient *θ* of ECP from the matrix of economic distance *W_e_*, the *θ* values in other models are all significantly negative, which suggests that there is a negative effect on spatial spillover in the value of ECP of geographically or administratively adjacent cities in the downstream area of YRB.

(1) In the weight matrix model of geographical distance *W_d_*, the convergence coefficient *β* of the ECP from the upstream, medium-stream, and downstream areas of the YRB is different, which makes the convergence speed different. The convergence speeds of the upstream, medium-stream, and downstream areas are 1.88%, 2.91%, and 3.67%, respectively. That is, the convergence speed of ECP in the downstream is the fastest, followed by the medium-stream, and the convergence speed of the upstream area is the slowest.

(2) In the weight matrix model of economic distance *W_e_*, the convergence rates of the upstream, medium-stream, and downstream areas are 2.00%, 3.23%, and 4.57%, respectively, that is, the convergence rates of ECP in the YRB from fast to slow are the downstream, medium-stream, and upstream area.

(3) In the administrative adjacent weight matrix model *W_a_*, the convergence rates of the upstream, medium-stream, and downstream areas are 1.95%, 3.43%, and 3.52%, respectively, that is, the convergence rates of ECP in the YRB from fast to slow are downstream, medium-stream, and upstream area.

(4) In the nested matrix model of economic and geographical distance *W_de_*, the convergence rates of the upstream, medium-stream, and downstream areas are 1.89%, 3.06%, and 3.76%, respectively, that is, the convergence rates of urban ECP in the YRB from fast to slow are the downstream, medium-stream, and upstream area.

On the whole, the convergence rate of ECP under different spatial weight matrices is consistent, and the downstream cities with a lower level of ECP have a higher convergence rate, while the upstream cities with a higher level of ECP have a lower convergence rate, which is also consistent with the convergence theory in neoclassical economic growth thought. In addition, comparing the convergence rate of ECP in the same basin under different spatial matrices, it can be discovered that the convergence rate of ECP in the upstream and downstream areas is relatively fast under the economic and geographical matrix. However, the convergence rate of ECP in the medium-stream is relatively fast under the weight matrix of administrative adjacency, which indicates that the degree of ECP has a high trend of coordination development from the upstream area and downstream area, while the degree of coordination development of ECP in the medium-stream area is relatively higher for administrative neighboring cities.

## 6. Conclusions and Policy Implications

Based on the city-panel data of 97 cities in YRB from 2003 to 2019, the index of ECP was calculated, and the spatial difference and convergence were tested by the Dagum Gini coefficient and spatial panel model. The results showed that:

(1) The index of ECP in YRB shows a steady growth trend, with an average growth rate of 4.71% yearly. The average Gini coefficient of ECP in the YRB indicates that the overall difference is low, and it has a downward trend. (2) The intra-regional differences of ECP from high to low are the medium-stream, the upstream, and the downstream area of YRB in turn. In different areas, the Gini coefficient between the medium-stream and downstream area is the largest (average value of Gini coefficient is 0.1561), with an average drop ratio of 3.11% yearly; the upstream and downstream is lowest, with a rate of 2.21%. (3) From the decomposition results of the regional whole differences of ECP in the YRB, the contribution of the density of transvariation is the highest with a rate of 43.37%, the intra-regional difference with a rate of 31.86%; the inter-regional difference with a rate of 24.77%. It indicates that the overall differences of ECP in YRB are getting smaller because of cooperation and governance, but the differences between and within regions exist because of geographical feature. (4) ECP has a significant spatial condition *β* convergence trend in upstream, medium-stream, and downstream area of YRB, and the convergence rate of ECP under different spatial weight matrices is consistent. (5) Under different spatial weight matrices, the convergence rate of ECP in upstream area and downstream area is relatively fast under the economic and geographical matrix. However, the convergence rate in the medium-stream is relatively fast under the weight matrix of administrative adjacency.

Based on the above conclusions, some policy implications and recommendations are as follows:

Firstly, the ECP level of the YRB displays a steady growth trend, the whole difference is low and it shows a downward trend, so the existing policy in the economy and environment is effective and needs to be upheld. In addition, along with the economic development and the change of residents’ demand, the policy in ecological protection (ecological civilization for a better environment of life) and high-quality development (common prosperity for a better quality of life) of YRB also needs to be adjusted by time, such as building the joint meeting of inter-provincial cooperation on ecological protection and high-quality development in the YRB, to protect the ecological environment, deepen scientific and technological innovation, enhance the supporting ability of innovation and cooperation, promote the integration of regional strategies, and expand the areas of open cooperation.

Secondly, the heterogeneity strategies on different provinces or areas should be formulated and adjusted from the view of environmental protection and high-quality development, due to the intra-regional and the inter-regional differences on ECP levels in the upstream, medium-stream, and downstream areas of YRB. Different provinces should make up their development strategies according to regional characteristics, and consider the influencing factors of ECP, such as climate, slope, temperature, precipitation, vegetation, economy, culture, and population. For example, in the upstream area of the YRB, which is rich in water and ecological resources, we should focus more on ecological vegetation protection, water resources protection, ecological safety, soil and water loss, climate control, eco-tourism, and other aspects. In the middle-stream area of the YRB, which is rich in soil and culture resources, we should focus more on the protection and development of the culture involving the history of the YRB, as well as wetland protection, tourism, ecological agriculture, forest and field security, etc. In the downstream area of the YRB, which is rich in water transport and industrial production, we should focus more on pollution control, energy consumption, air quality, ecological leisure, and ecological industry development. At the same time, different regions should strengthen their connection with economic, geographical, and administrative neighboring cities to realize coordination in ECP as partners.

Thirdly, given the obvious spatial difference and convergence in the coordination of ECP, so it is necessary and worthy to strengthen communication and cooperation between regions or areas, and put into effect on coordinated governance of cross-regional environmental pollution, as well as conduct the multi-regional economic coordination and cooperation, such as establishing environmental and ecological compensation mechanism; the upstream provides water, air, and other good ecological resources for the middle-stream and downstream area of YRB, and the downstream provides financial and industrial support for ecological protection and governance for the upstream or middle-stream. Moreover, they can cooperate in policy and regulation on emission reduction and carbon reduction, water efficiency, the greening program, and integrated protection and restoration project of landscape, forest, farmland, lake, grass, and sand.

Realizing complementary advantages economically, geographically, and administratively based on different resource endowments and development stages in the upstream area, medium-stream area, and the downstream area of YRB is more beneficial to achieve a better environment and a better quality of life for all people, as well as to achieve the long-term goals of socialist modernization in 2035, and the goals of “carbon peak” in 2030 and “carbon neutral” in 2060.

## Figures and Tables

**Table 1:** The index system of ECP.

Index	Indicators				Third-Level Indicators	Weight	Meaning of Basic Indicator	Value Reflected by Indicators
	First-Level Indicators	Weight	Second-Level Indicators	Weight				
The index System of ECP	Set up the concept of ecology when the society develops fast and becomes rich together.	0.471	Ecological economy	0.809	GTFP	0.290	Green total factor productivity based on input-output perspective.	Social and economic efficiency
					Patent	0.485	Application quantity of green practical patent; Application quantity of green invention patent.	Future development potential
					Wealth	0.225	Urban GDP per capita (yuan); Per capita disposable income of residents (yuan); Engel coefficient in disposable income of urban and rural residents.	Social common prosperity
			Ecological management	0.191	Ecological management	0.387	Comprehensive utilization rate of industrial waste (%); Urban sewage treatment rate (%); Harmless treatment rate of domestic garbage (%).	Economic governance ability
					Pollution control	0.613	Industrial smoke and dust emissions (tons); Wastewater discharge (10,000 tons); Sulfur dioxide emissions (tons).	
	Establishing the concept of common in the construction of ecological civilization	0.529	Ecological environment	0.117	Environment	0.681	Concentration of PM2.5; energy consumption per unit GDP.	Common ecological environment
					Ecology	0.319	Including regional water resources; geographical slope; sunshine duration; regional average temperature.	Common ecological quality
			Ecological resources	0.883	Industry	0.364	Gross output value of agriculture; forestry and fishery (100 million yuan). Domestic tourism revenue (millions);	Ecological resources development potential
					Nature	0.636	Area of nature reserve	Common Ecological sharing

Note: The index of ECP was obtained based on two first-level indicators by the entropy method; first-level indicators were obtained based on four second-level indicators by the entropy method; second-level indicators were obtained based on nine third-level indicators by the entropy method; third-level indicators by weighting were obtained based on 21 basic indicators by the entropy method. In order to ensure the robustness of the entropy method, the paper also uses the principal component analysis method to calculate the index of ECP. The Pearson correlation coefficient between them (the ECP index by different methods) is 0.7782, which statistically satisfies the significance level of 1%, which fully shows that the results and the entropy method are robust. Relevant scholars’ research on the third-level indicators and index selection reasons from the scholars’ research is not listed, it can be obtained from the author.

**Table 2:** The description of variables.

Variables	Description	Min.	Max.	Mean	Std. Dev.	Obs
*GRE*	The logarithmic growth rate of ECP	−0.284	0.429	0.048	0.071	1552
*ECP*	Index of ecological common prosperity	0.135	0.880	0.371	0.132	1552
*PD*	Population density (log)	2.281	8.567	5.572	1.127	1552
*PGDP*	Real GDP per capita (log)	7.545	12.230	9.837	0.835	1552
*IS*	Industrial structure	0.182	0.849	0.507	0.115	1552
*SA*	Specialization agglomeration	1.240	17.210	3.418	2.341	1552
*DA*	Diversified agglomeration	0.889	7.477	2.272	0.928	1552
*ER*	Environmental regulation	0.139	0.916	0.702	0.163	1552
*TI*	Technology investment	0.059	6.258	0.865	0.733	1552
*HC*	Human capital	−4.806	7.179	4.135	1.584	1552

**Table 3:** Moran Index of ECP in the YRB from 2003 to 2019.

Year	*W_d_*		*W_e_*		*W_a_*		*W_de_*	
	I Value	Z Value	I Value	Z Value	I Value	Z Value	I Value	Z Value
2003	0.142 ***	10.547	0.067	1.376	0.621 ***	15.382	0.128 ***	6.946
2004	0.149 ***	11.045	0.089 *	1.758	0.652 ***	16.107	0.136 ***	7.307
2005	0.151 ***	11.112	0.094 *	1.849	0.621 ***	15.270	0.137 ***	7.341
2006	0.152 ***	11.212	0.095 *	1.859	0.610 ***	15.021	0.139 ***	7.429
2007	0.137 ***	10.154	0.088 *	1.730	0.561 ***	13.843	0.124 ***	6.691
2008	0.118 ***	8.865	0.148 ***	2.793	0.489 ***	12.092	0.114 ***	6.197
2009	0.115 ***	8.639	0.168 ***	3.140	0.440 ***	10.893	0.119 ***	6.426
2010	0.111 ***	8.344	0.185 ***	3.440	0.403 ***	9.991	0.114 ***	6.156
2003	0.101 ***	7.662	0.199 ***	3.685	0.356 ***	8.856	0.109 ***	5.940
2012	0.116 ***	8.658	0.203 ***	3.769	0.387 ***	9.593	0.122 ***	6.567
2013	0.121 ***	9.036	0.252 ***	4.622	0.342 ***	8.503	0.133 ***	7.127
2014	0.121 ***	9.058	0.228 ***	4.207	0.389 ***	9.656	0.135 ***	7.213
2015	0.139 ***	10.285	0.254 ***	4.661	0.387 ***	9.597	0.151 ***	8.001
2016	0.118 ***	8.837	0.273 ***	4.990	0.345 ***	8.583	0.138 ***	7.346
2017	0.118 ***	8.837	0.235 ***	4.331	0.306 ***	7.669	0.136 ***	7.277
2018	0.122 ***	9.112	0.218 ***	4.043	0.285 ***	7.157	0.138 ***	7.370
2019	0.120 ***	9.010	0.196 ***	3.649	0.304 ***	7.603	0.135 ***	7.204

Note: *** and * are significant at the level of 1% and 10%, respectively.

**Table 4:** Estimated results of the global spatial convergence model for ECP in the YRB.

Variable	*W_d_*	*W_e_*	*W_a_*	*W_de_*
*β(ln ECP)*	−0.310 ***	−0.325 ***	−0.341 ***	−0.311 ***
	(0.019)	(0.019)	(0.020)	(0.019)
*θ(w × ln ECP)*	0.203 **	0.024	0.228 ***	0.150 *
	(0.084)	(0.044)	(0.035)	(0.080)
*ρ*	0.678 ***	0.259 ***	0.454 ***	0.610 ***
	(0.058)	(0.040)	(0.035)	(0.061)
Control variable	YES	YES	YES	YES
Sample size	1455	1455	1455	1455
R^2^	0.194	0.196	0.173	0.205
Log-likelihood	1962.267	1931.441	1961.654	1959.786
Convergence rate	2.32	2.46	2.61	2.33

Note: ***, **, and * are significant at the level of 1%, 5%, and 10%, respectively, and the standard errors are in brackets.

**Table 5:** Estimation results of regional spatial convergence model for ECP in the YRB.

Region	Variable	*W_d_*	*W_e_*	*W_a_*	*W_de_*
Upstream in YRB	*β (ln ECP)*	−0.260 ***	−0.274 ***	−0.268 ***	−0.261 ***
		(0.031)	(0.031)	(0.032)	(0.031)
	*θ (w × ln ECP)*	0.087	0.059	0.145 **	0.132
		(0.115)	(0.066)	(0.060)	(0.110)
	*ρ*	0.261 **	0.013 *	0.182 **	0.227 **
		(0.112)	(0.003)	(0.079)	(0.113)
	Control variable	YES	YES	YES	YES
	Sample size	525	525	525	525
	R^2^	0.196	0.202	0.198	0.189
	Log-likelihood	772.465	772.753	774.334	770.356
	Convergence rate v	1.88	2.00	1.95	1.89
Medium-stream in YRB	*β (ln ECP)*	−0.372 ***	−0.404 ***	−0.422 ***	−0.387 ***
		(0.037)	(0.036)	(0.038)	(0.037)
	*θ (w × ln ECP)*	0.311 ***	0.119 *	0.299 ***	0.342***
		(0.120)	(0.071)	(0.070)	(0.115)
	*ρ*	0.386 ***	0.142**	0.308 ***	0.379 ***
		(0.099)	(0.062)	(0.072)	(0.098)
	Control variable	YES	YES	YES	YES
	Sample size	435	435	435	435
	R^2^	0.225	0.254	0.248	0.231
	Log-likelihood	566.911	569.392	573.079	569.283
	Convergence rate v	2.91	3.23	3.43	3.06
Downstream in YRB	*β (ln ECP)*	−0.444 ***	−0.519 ***	−0.431 ***	−0.452 ***
		(0.036)	(0.038)	(0.036)	(0.036)
	*θ (w × ln ECP)*	−0.419 ***	0.177 ***	−0.267 **	−0.112 **
		(0.158)	(0.066)	(0.128)	(0.026)
	*ρ*	0.333 ***	0.408 ***	0.407 ***	0.454 ***
		(0.099)	(0.048)	(0.076)	(0.082)
	Control variable	YES	YES	YES	YES
	Sample size	495	495	495	495
	R^2^	0.397	0.328	0.368	0.374
	Log-likelihood	684.539	682.985	681.065	682.156
	Convergence rate v	3.67	4.57	3.52	3.76

Note: ***, **, and * are significant at the level of 1%, 5%, and 10%, respectively, and the standard errors are in bracket.

## Data Availability

Information Network of Development Research Center of the State Council of China, China Urban Statistical Yearbook.
